# Note on the Relations between Syphilis and Locomotor Ataxy

**Published:** 1881-12

**Authors:** Julius Althaus


### Note on the Relations Between Syphilis and Locomotor Ataxy.

By Julius Althaus, M. D., M. R. C. P.

The question whether syphilis should be looked upon as a frequent, or indeed the principal, cause of locomotor ataxy, has recently been much discussed, most of the French and English observers being strongly in favor of this view, while our German *confreres* have expressed the contrary opinion. I therefore wish to add some of my own experience in the etiology of ataxy to the facts already published, and have with this view analyzed a thousand consecutive cases of nervous affections which have been under my care in private practice, with the object of discovering the part which syphilis has played in their causation. Amongst these thousand cases there were—206 of epilepsy; 101 of neurasthenia, or nervous exhaustion, without evidence of substantial lesions of the nervous system; 77 of hemiplegia owing to cerebral hæmorrhage or softening; 51 of neuralgia; and 32 cases of ataxy with fully developed symptoms; the remainder of the cases being such as hysteria, infantile paralysis local paralysis, muscular atrophy, anæsthesia, chorea, tumor of the brain, impotency, paralysis agitans, torticollis, etc. In 29 out of 32 cases of ataxy there was a syphilitic history; and in these 29, secondary symptoms had occurred in 28, while in one of them there had been a soft chancre and a bubo, but no secondaries.

These results are certainly startling, as they show a percentage of 90.6 in favor of the syphilitic origin of tabes dorsalis, which is higher than that found by Gowers (70) and Erb (67.) They become even more striking on being compared with the percentages found for the other nervous affections, inasmuch as of 206 cases of epilepsy only 10, of 101 cases of neurasthenia only 12, of 77 cases of hemiplegia 5, and of 51 cases of neuralgia only 2, had been preceded by syphilis. The percentages are therefore as follows:

| | | | | |
|---|---|---|---|---|
| Ataxy | was preceded | by syphilis | in 90.6 | per cent. |
| Neurasthenia | " | " | 11.8 | " |
| Hemiplegia | " | " | 6.2 | " |
| Epilepsy | " | " | 4.8 | " |
| Neuralgia | " | " | 3.9 | " |

There were 6 additional cases in which paralysis of the ocular muscles, shooting pains, and sexual debility rendered it probable that sclerosis was developing in the posterior columns of the cord, and in 4 of these there were syphilitic antecedents. These cases, however, were lost sight of before the symptoms had become unequivocal; and, as they all occurred some time before the loss of the patellar tendon-reflex was utilized for the diagnosis of this disease, I think it better to exclude them. Yet it is a significant fact that in 4 out of these 6 doubtful cases syphilis should have previously occurred, giving a percentage of 66.6. If we were to add them to the fully developed cases, the percentage of the whole would amount to 86.8.

With regard to the interval which had elapsed between the first symptoms of syphilis and of tabes, it was found that the former had preceded the latter upwards of twenty years in 2 cases, between ten and twenty years in 7 cases, between two and ten years in 19 cases, and eighteen months in 1 case. Amongst the 3 cases in which there was no history of syphilis, the affection was attributed in one to an operation for piles, in another to an accident in a tramcar, and in a third to exposure to wet and cold. The age at which ataxy became developed was from twenty-one to forty-five, and all the patients were males. It is to this latter circumstance that I attribute the higher percentage of syphilitic antecedents in my cases, as ataxy in women is generally not preceded by syphilis. In all my cases, one or another of the ordinary causes of ataxy, such as accidents, over-exertion, combined with the influence of wet and cold, and sexual or alcoholic excesses, were mentioned as having led to the outbreak of the complaint.

An overwhelming numerical testimony is thus clearly exhibited in favor of the view that tabes is habitually preceded—I do not say produced—by syphilis. But the real question at issue is whether syphilis is the originator of ataxy, or merely an accidental concomitant of it. The length of time which had generally intervened between the outbreak of the two diseases does not speak against their standing in the relation of cause and effect. Sir Benjamin Brodie met with an accident while riding on horseback in 1834, dislocating his right shoulder, and he eventually died of cancer which had become developed in the same joint in September, 1862, which gives an interval of twenty-eight years between the injury and the consecutive disease. It would not be difficult to multiply such examples. Other considerations, however, serve to show that ataxy is a disease, *per se*, which occurs without any syphilitic taint whatever, but which may, like so many other disease, be imitated by syphilis under certain circumstances.

1st. Tabes has unquestionably existed in Europe long before the first appearance of syphilis in this quarter of the globe.

2d. Numerous cases of ataxy are on record, more especially as having occurred in women, where no syphilitic affection whatever had preceded it.

3d. Ataxy is not a common or inevitable consequence of syphilis, as, for instance, roseola or sore-throat, but only appears to become developed in the syphilitic, if they have the neurotic constitution, and if other causes, such as accidents, excesses, and the influence of wet and cold, etc., have also been active.

4th. Treatment by iodide of potassium, even if used perseveringly and in large doses, is only exceptionally useful, even in cases with a pronounced syphilitic history, while the exhibition of nitrate of silver, ergot, the continuous galvanic current, nerve-stretching, and general remedial measures which have the tendency to improve the condition of the central nervous system, but which have no influence on the syphilitic diathesis, are frequently productive of beneficial results.

5th. The same disease—sclerosis—occurs in the lateral column of the spinal cord, more particularly in young women who have never been exposed to venereal infection.

6th. Syphilis may, however, imitate ataxy (to use an expression of Mr. Johnathan Hutchinson), just as it imitates iritis, lupus, rodent ulcer, and other diseases. Clinically the symptoms of syphilitic and non-syphilitic tabes appear to be identical. That ataxy should be so frequently associated with syphilis is probably owing—first, to the fact that syphilis, like masturbation and other excesses and irregularities, deteriorates the nutrition and power of resistance of the central nervous system; and second, and more particularly to the specific tendency of the syphilitic virus, to lead to low forms of local inflammation, which, when once established, are apt to spread in certain definite paths. Where this kind of inflammation attacks the posterior root-zones of the spinal cord, it will produce the clinical aspect of locomotor ataxy, being aided in its development and further progress by the natural tendency of spinal disease to invade symmetrical and homologous anatomical and histological portions of the organ:—*London Lancet.*

